# Vision screening and vocational aptitude: A factor analysis approach

**DOI:** 10.1371/journal.pone.0286513

**Published:** 2023-05-31

**Authors:** Eric S. Seemiller, James Gaska, Eleanor O’Keefe, Elizabeth Shoda, Jonelle Knapp, Marc Winterbottom

**Affiliations:** 1 Air Force Research Laboratory, 711th Human Performance Wing, Wright-Patterson Air Force Base, Dayton, OH, United States of America; 2 KBR, inc., Beavercreek, OH, United States of America; The Ohio State University, UNITED STATES

## Abstract

For a good vision screening battery to quickly and accurately reflect the status of the human visual system it should be relevant, reliable, and streamlined. Because the early visual system has limited functional architecture, many simple measurements of the visual system may in fact be measuring the shared computations and parallel processes of other visual functions, making much of the measurement process redundant. This can make a screening battery repetitious and therefore inefficient. The purpose of this research is to investigate these redundancies in a large occupational screening dataset using factor analysis. 192 subjects participated in the Operational Based Vision Assessment (OBVA) Laboratory Automated Vision Testing (AVT) procedure. The AVT includes digital tests for visual acuity, luminance and cone contrast sensitivity, motion coherence, stereopsis, and binocular motor function. Psychometric thresholds and fusional ranges were collected from each subject and a factor analysis was utilized to investigate independent latent variables in the dataset. A *promax* rotation revealed 5 factors that explained 74% of the total variance: (1) medium and high spatial frequency vision, (2) stereoacuity and horizontal fusional range, (3) cone contrast sensitivity, (4) motion perception, and (5) low spatial frequency vision. Practically, these results suggest that the screening battery can be reduced to 5 independent measurements that capture much of the variance in the dataset. Furthermore, the factors predicted operational and vocational aptitude better than any single variable. More interestingly, these relationships also reiterate known computational processes within the human visual system, such as the parallel processing of low and high spatial frequency content.

## Introduction

Vision screening batteries should quickly provide a reliable and holistic snapshot of the visual system. The Automated Vision Tester (AVT), developed by the Operational Based Vision Assessment Laboratory (OBVA; United States Air Force), is a stand-alone digital vision testing unit that employs a battery of sensory and motor visual function tests with the goal of modernizing visual medical screening standards for Airmen entering the United States Air Force (USAF) with increased resolution and quantitative utility. While current analog screening tools include memorizable tests with poor test-retest reliability and other quantitative limitations, previous technical reports have reported on the validity and reliability of the tests contained within the AVT, demonstrating an improvement in some of these limitations [1–3]. The purpose of the investigation reported here is to further streamline the AVT battery and provide maximum efficiency for characterizing an individual’s visual system and predicting ecologically relevant visual performance. The AVT has been tested in multiple laboratories and provides a unique data set with accurate and reliable vision tests to support this analysis [2].

Because the human visual system contains limited functional architecture and all visual information enters the visual system through the same early pathways, there are often shared computations and processes. Most vision tests do not directly test any one of these pathways, but rather they test the sum total of a sensory process. As a result, the output of a particular vision test is likely only loosely correlated to an underlying visual process. A quantitative, multivariate approach called factor analysis can be used to identify the latent correlations between measured variables and true underlying factors [4,5], such as visual pathways.

Factor analysis is increasingly being used in vision science to identify and characterize factors of visual function related to measured sensory variables. For example, McKee et al. [6] demonstrated that observed patterns in conventional testing of visual deficit in human amblyopes were actually well correlated to two underlying factors: acuity and contrast sensitivity. Peterzell et al. [7–9] utilized a similar approach to highlight this phenomenon, showing that testing contrast sensitivity across a number of spatial frequencies (SF) identified essentially two underlying components of spatial vision: high and low SF vision. Other investigations have demonstrated the utility of factor analysis in similar sensory vision datasets related to binocular vision [10,11], color vision [12,13] and general visual performance [14–16].

Applying factor analysis to data obtained using the AVT has two goals: (1) identify and eliminate redundancies and (2) identify operationally or ecologically relevant vision metrics. Identifying redundancies within the AVT screening dataset can help streamline the test battery, ultimately decreasing the time needed for USAF vision screening. That is, if multiple variables are well-correlated with only one factor, the number of variables needed to assess visual function can be reduced. This may substantially reduce the time it takes for the AVT to take a thorough snapshot of the individual’s visual system. Secondly, it may also be of greater utility for the AVT (or any vision screening battery) to identify more operationally or ecologically relevant vision factors than any single measured variable. The identified factors may be more useful than the individual components.

Here we report the results of an exploratory factor analysis on a large vision screening dataset. Using 14 normalized variables taken from the AVT battery, we identify five underlying vision factors. Furthermore, we demonstrate that two factors are better at distinguishing performance for individuals within a specific USAF career field, in this case air refueling boom operators, vs. individuals outside that career field, in comparison to any single variable measured, implying its utility for predicting operationally relevant visual performance.

## Methods

### Subjects

This work adhered to the tenets of the Declaration of Helsinki. We recruited 171 subjects (101 male, mean age ± SD = 41.08 ±14.1) from the Dayton, OH area, along with 21 USAF active-duty boom operators (20 male, mean age ± SD = 36.2 ± 8.9). There was no statistically significant difference between the ages of the two groups. All participants provided written informed consent and participants were either compensated for their participation or, for USAF participants, volunteered their time. This research was carried out at the 711 HPW/RHBC Operational Based Vision Assessment (OBVA) Laboratory in accordance with IRB approved research protocols F-WR-2017-0095-H and FWR-2013-0074.

### AVT

**Fig 1:**
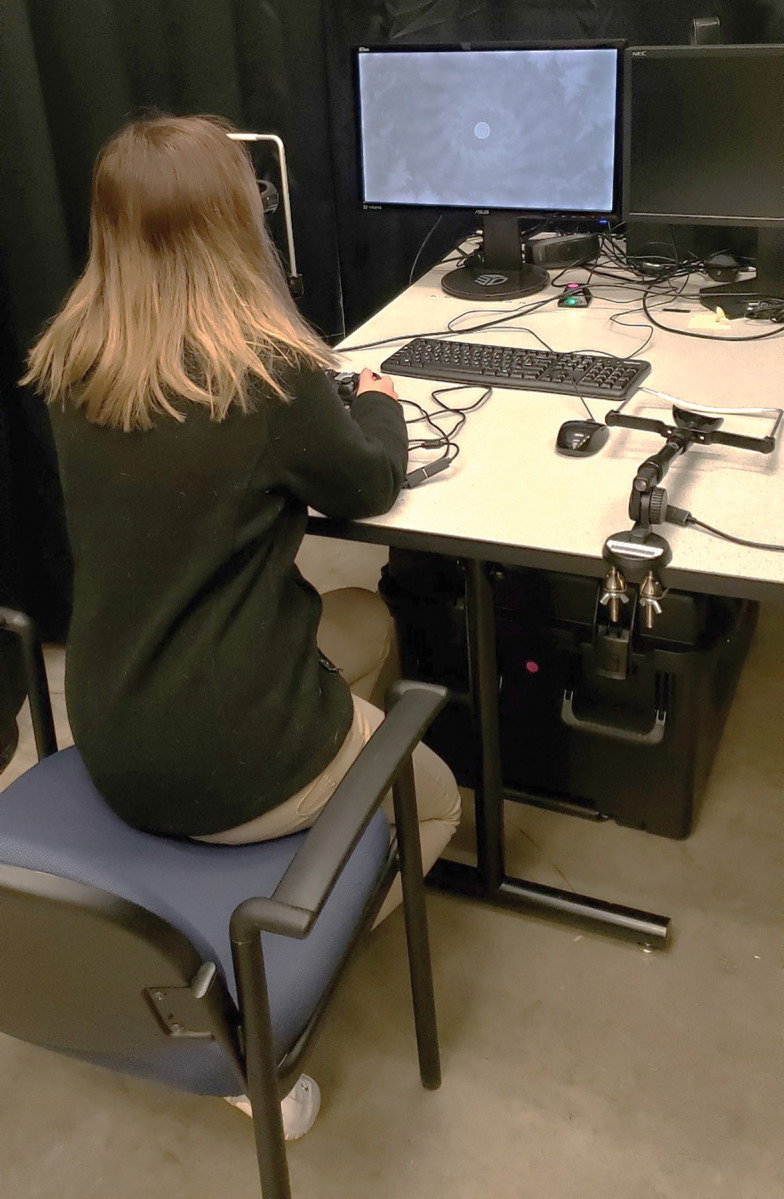
The automated vision tester. Dual-ring stereo test (1 m) shown here.

The AVT (Fig 1) is a computer-based vision testing system developed by the OBVA Laboratory. It consists of a Windows-based PC (HP Pavilion Gaming Desktop 690–00, HP, Palo Alto) and two monitors. The first is a 27-inch Asus VG278HE 3D monitor (AsusTek Computer Inc., Taiwan) with a spatial resolution of 1920 x 1080 and a frame rate of 120 Hz. It is compatible with Nvidia 3D Vision2 active shutter glasses (Nvidia Corporation, Santa Clara, California) for dichoptic presentation, which allows for separate images to be shown to the two eyes. The second monitor, a 23- inch LCD NEC Multisync P232W (NEC Corporation, Tokyo) with a resolution of 1920 x 1080 pixels, is used primarily for luminance and color contrast sensitivity testing. The AVT tests were administered at either 1 m or 4 m. The AVT battery included tests for visual acuity, contrast sensitivity, cone contrast sensitivity, stereo acuity, fusion range and motion coherence. All testing occurred in a darkened room, though no steps were taken to match room illuminance across all sessions and subjects.

### Spatial vision tests

**Fig 2:**
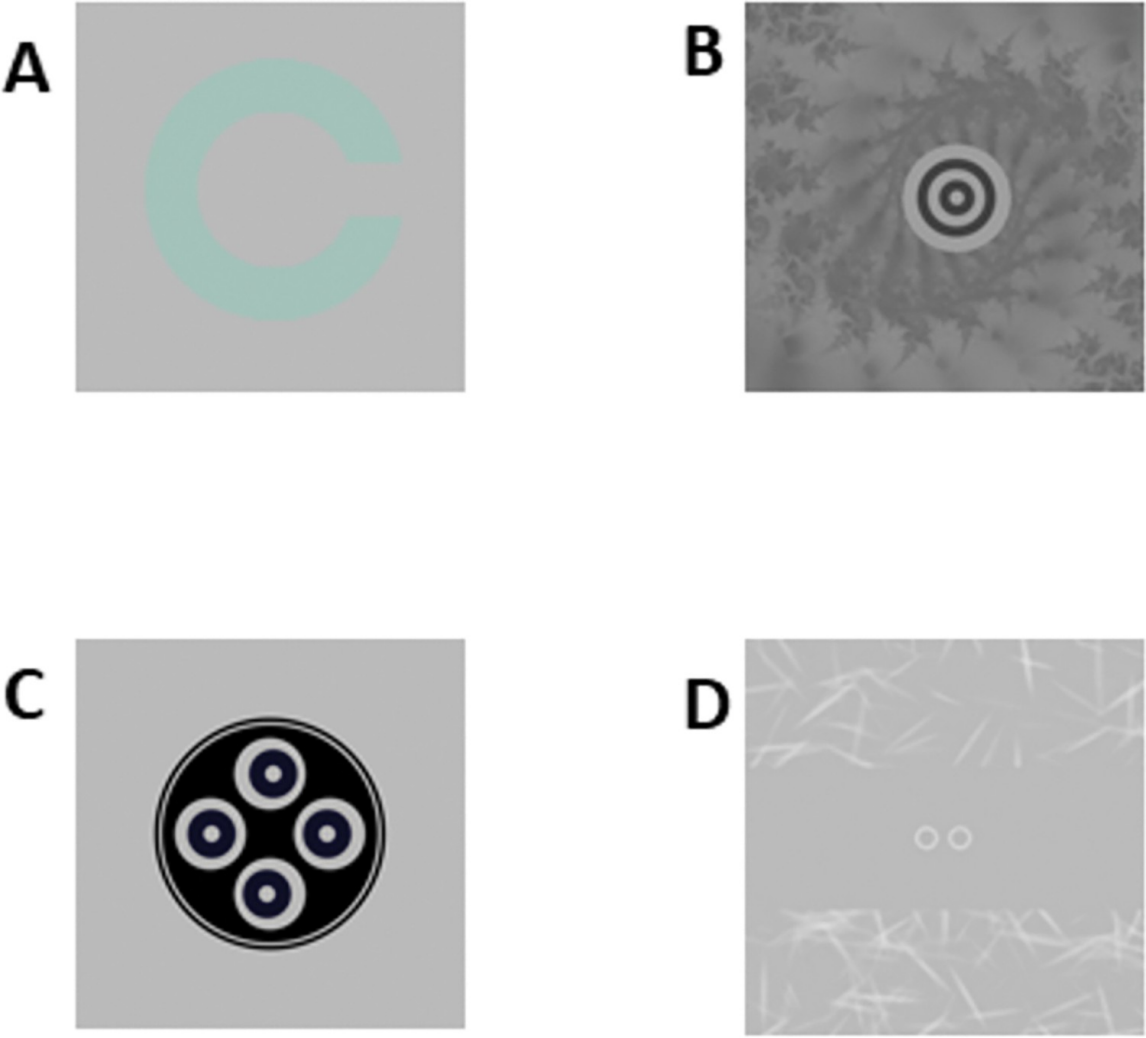
AVT stimuli. A. Landolt C stimulus used for achromatic contrast sensitivity, visual acuity, and cone contrast sensitivity (M-cone contrast sensitivity shown here). B. Dual-ring stereopsis. The inner ring appears in front of or behind the outer ring. C. Stereo-search test (SST). One ring appears in front of the screen, one ring appears behind the screen, and the other two are at the plane of the screen. D. Fusion range. The two rings, presented dichoptically, move either toward or away from one another until the subject reports diplopia (horizontal fusion range shown here).

Spatial vision testing consisted of visual acuity and achromatic contrast sensitivity (ACS) were measured at 4 m. ACS was measured with Landolt C’s (Fig 2A) of three sizes: 83.33, 12.5 and 6.25 arcmin, corresponding to a gap of 16.7, 2.5 and 1.25 arcmins. These correspond to 20/333, 20/50 and 20/25 Snellen letter sizes (or 1.8, 12 and 20 cycles per degree). For visual acuity, the size of the Landolt C optotype changes on each trial. For ACS, the contrast changes on each trial. The subject is asked to identify the location of the gap in a 4 alternative-forced choice (AFC) paradigm. Thresholds were measured using the psi method [17] and the Palamedes Toolbox [18]. This is an adaptive procedure that selects the most informative stimulus amplitude for each trial using a Gumbel (log-Weibull) function. We used a 40-trial block, allowing slope and threshold (70.8% correct) parameters to vary, while fixing the gamma (guess rate) and lambda (lapse rate, or finger-error rate) parameters. For acuity, the threshold was the smallest size of the optotype the observer could resolve, and for ACS, the threshold was the lowest Weber contrast.

### Stereopsis tests

The AVT employs dual-ring disparity discrimination tests (Fig 2B). This consists of two concentric circles. The larger circle has zero disparity while the smaller inner circle has either crossed or uncrossed disparity. In a 2AFC paradigm, the subject is asked whether the smaller circle appears in front of or behind the larger circle or to identify the sign of the disparity. The same Psi thresholding procedure is used as above, and the threshold is the smallest disparity of which the subject could discriminate the sign. This test was performed at both 1m (near) and 4m (far). A third test of disparity discrimination, called stereo-search (Fig 2C), was also used. This consists of four circles: two with zero-disparity, one with crossed, and one with uncrossed. In a 4AFC paradigm, the subject is asked to identify the circle that appears in front of the screen (top, bottom, right, or left). This test is performed at 4m.

### Color vision tests

Cone contrast sensitivities (CCS) were measured using the NEC monitor described above. This procedure is described in detail elsewhere [1]. Briefly, contrast sensitivity is measured using an 83.33 arcmin Landolt C (equivalent to 20/333 Snellen letter size), following a color-space transformation that isolates chromatic information as a function of expected cone excitation [19,20]. That is, for each cone type, it measures the discriminable change in cone excitation in the optotype from the background cone excitation (ΔL/L, ΔM/M, and ΔS/S), while holding other cone excitations fixed. The psychometric procedure is identical to the ACS procedure. It is worth noting that these metrics do not address and may be somewhat independent from higher order color perception, such as hue perception.

### Motion coherence

We assessed global motion perception by testing radial and rotational motion coherence, similar to paradigms employed in previous research [21]. For radial motion, a field of dots moved either toward or away from a focus of expansion in the middle of the field. Subjects were asked to determine the direction in a 2AFC paradigm. The percentage of dots moving coherently was varied on each trial. The threshold was the percentage of coherent dots for which one could reliably determine the direction of motion and estimated as the alpha of the best fit Gumbel function (similar to the above tests). Rotational motion was measured the same way, except dots rotated clockwise or counterclockwise.

### Fusional range

Fusional ranges were measured by asking participants to report when a binocular circular target could no longer be fused (Fig 2D). As such, it is the only truly subjective test within the screening battery. The target increased in disparity at a velocity of 0.62°/s (vertical) 4.96°/s (horizontal). The subject pressed a button when the target could not be fused. The same procedure was repeated with the disparity becoming smaller. The subject pressed a button when the target could be re-fused. Each of these was measured three times. The average recovery ranges were calculated for both crossed and uncrossed and added for a total horizontal fusion range. Similarly, vertical recovery ranges were averaged for both right eye up and right eye down directions.

### Factor analysis

To assess the dataspace, thresholds from the AVT were first log transformed and standardized into normal distributions. Multivariate data analyses were performed using MATLAB Statistics Toolbox (The Mathworks, Natick MA). First, a principal components analysis was employed, which suggested that there are 5 components with eigenvalues greater than 1. Second, a factor analysis was performed, using MATLAB’s *factoran* function, searching for 5 factors using the promax rotation [22–24]. Promax is a computationally faster type of direct oblimin rotation, in which factors can be correlated with one another. We chose to allow oblique rotation of factors in order to represent relationships between parallel processes within the visual system. Indeed, if one wanted to analyze purely independent factors, an orthogonal rotation would be more appropriate. However, previous pilot data reported elsewhere [25] suggest good agreement between rotational strategies in this dataset. Bootstrapped confidence intervals for factor loadings were iterated across 10,000 samples.

**Fig 3:**
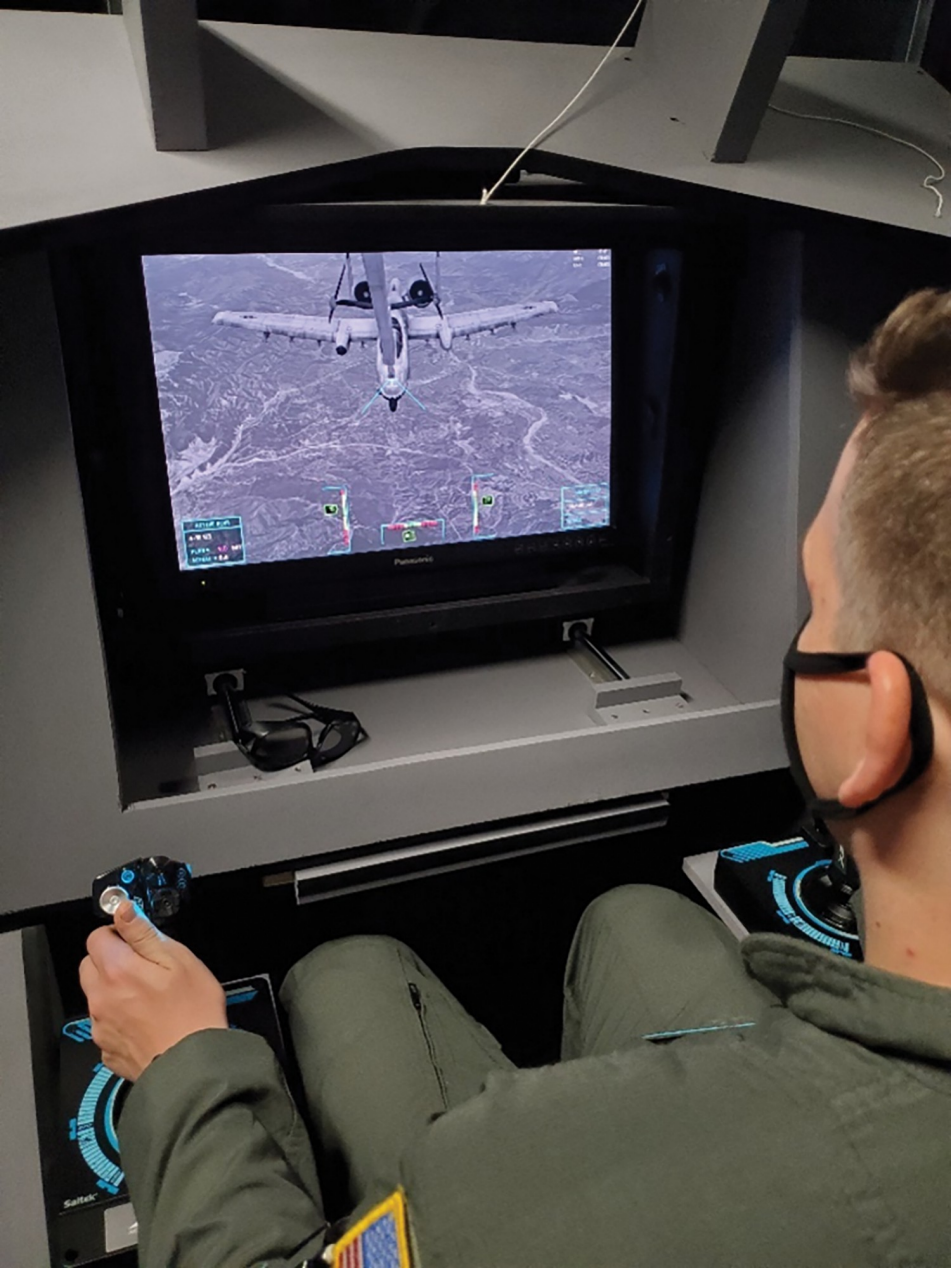
A USAF boom operator operating an aerial refueling simulation.

Included in the dataset were AVT results from 21 USAF In-flight Refueling Specialists, commonly known as boom operators or booms (Fig 3). Boom operation or aerial refueling operation requires Airmen to fly a refueling boom located at the rear of a tanker aircraft and guide it into the receptacle on a receiver aircraft located approximately 15 m away. For most aircraft this requires extreme precision, and it is thought that USAF boom operators may require superior spatial and binocular vision in order to perform this task safely and reliably. USAF Medical standards require Boom operators have the following criteria: BCVA of 20/20 at distance and near, < = 3.0D astigmatism, < = 5.5D refractive error in any meridian, < = 3.5D anisometropia in any meridian, < = 10 PD of lateral phoria, < = 1.5 PD of vertical phoria, 40 arcseconds of stereoacuity and normal L, M and S cone contrast sensitivity [26]. We wanted to determine if multivariate factors within our data set could better discriminate between booms and non-booms better than individual measures of vision. To do this, we calculated Cohen’s D effect size [27] between the boom and non-boom group means for each individual variable and all 5 factors. Cohen’s D is simply the difference between the means of two populations divided by the pooled standard deviation, comparable to a Z-score.

## Results

**Fig 4:**
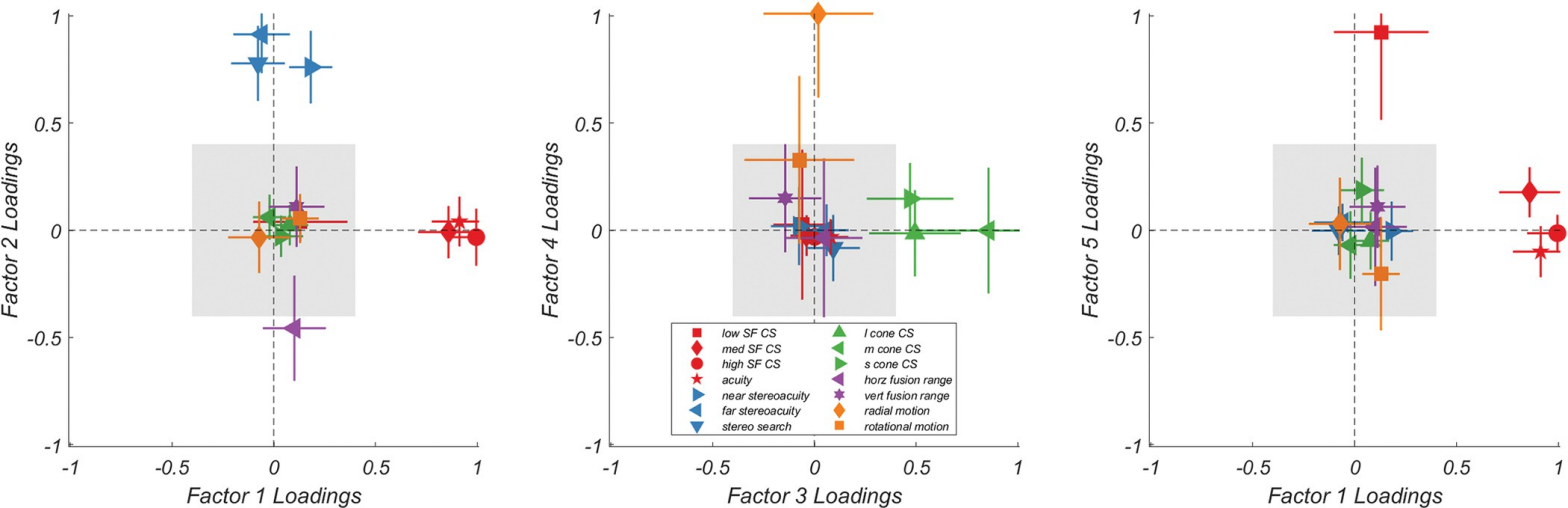
Factor loadings for all five factors. Left) Factors 1 & 2, Middle) Factors 3 & 4, and Right) Factors 1 & 5. Variables are color-coded based on investigator predicted variable type. Error bars are 95% bootstrapped confidence intervals. The grey bars represent an estimate of spurious correlation; variables that load in this range are unlikely to be strongly related to the underlying factor.

A principal component analysis revealed five factors with an eigenvalue greater than 1. We therefore ran a factor analysis for five factors that accounted for 82% of the variance in the dataset. The loadings of these factors are plotted in Fig 4. Factor 1 (Fig 4, Left) loaded heavily on 3 out of 4 spatial vision variables (including medium and high SF CS along with visual acuity). Low SF CS remained uncorrelated to Factor 1. The second factor (Fig 4, Left) loaded heavily on variables related to binocular vision, including all 3 tests of stereopsis. Horizontal fusional range was also inversely correlated, suggesting that as one performed poorly on stereopsis (i.e., they had high stereoacuity thresholds), their horizontal fusional range also decreased. Conversely, if one has good stereopsis related to Factor 2, they also have a large horizontal fusional range. Interestingly, Factor 2 appears unrelated to vertical fusional range.

Factor 3 (Fig 4, Middle) was related to color vision and was highly correlated with long-, medium-, and short- wavelength cone contrast sensitivity. Factor 4 (Fig 4, Middle) loaded strongly on rotational motion coherence and was only marginally correlated to radial motion coherence. Finally, Factor 5 (Fig 4, Right) was driven relatively independently by low SF CS and was unrelated to any other variable, including the other spatial vision variables.

**Fig 5:**
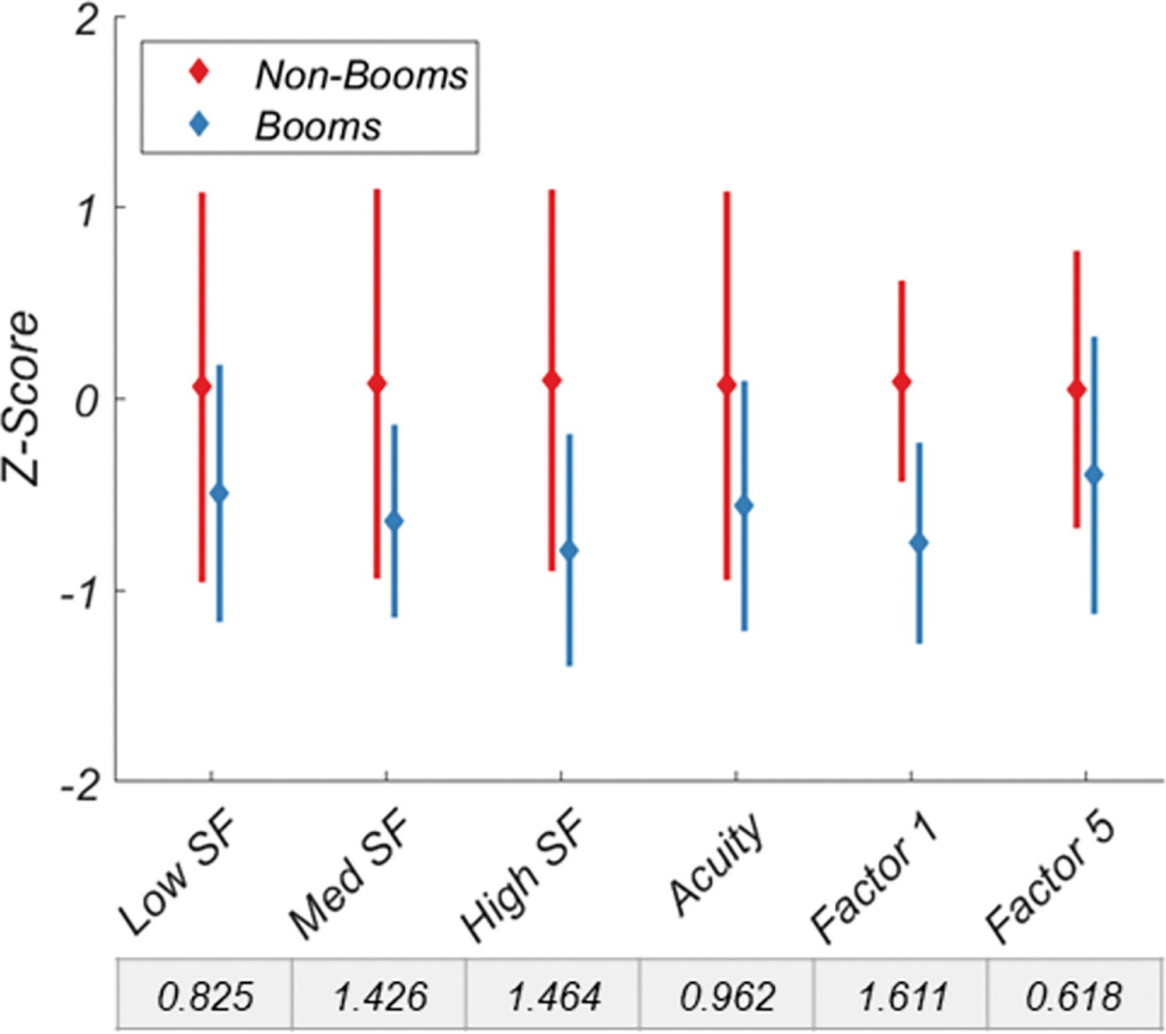
Discriminability of non-booms and booms for the spatial vision variables and factors 1 and 5. Data are normalized. Cohen’s D for each variable and factor is shown below the x-axis labels. Error bars are +/- 1 SD.

We were also interested in how well factors could predict group discrimination in a visually relevant career field, in this case boom operators. We compared the factor scores and individual variable scores of 21 boom operators (included in the factor rotation) and the other 171 non-boom operators in the dataset. Fig 5 shows the discriminability between the two groups for their normalized individual variables and factor scores for Factors 1 and 5, the spatial vision factors. Though 2 out of 4 spatial vision variables (medium and high SF CS) discriminated the two groups well (i.e., Cohen’s D > 1.0) none performed as well as Factor 1. Factor 5 performed the worst of the spatial vision variables.

**Fig 6:**
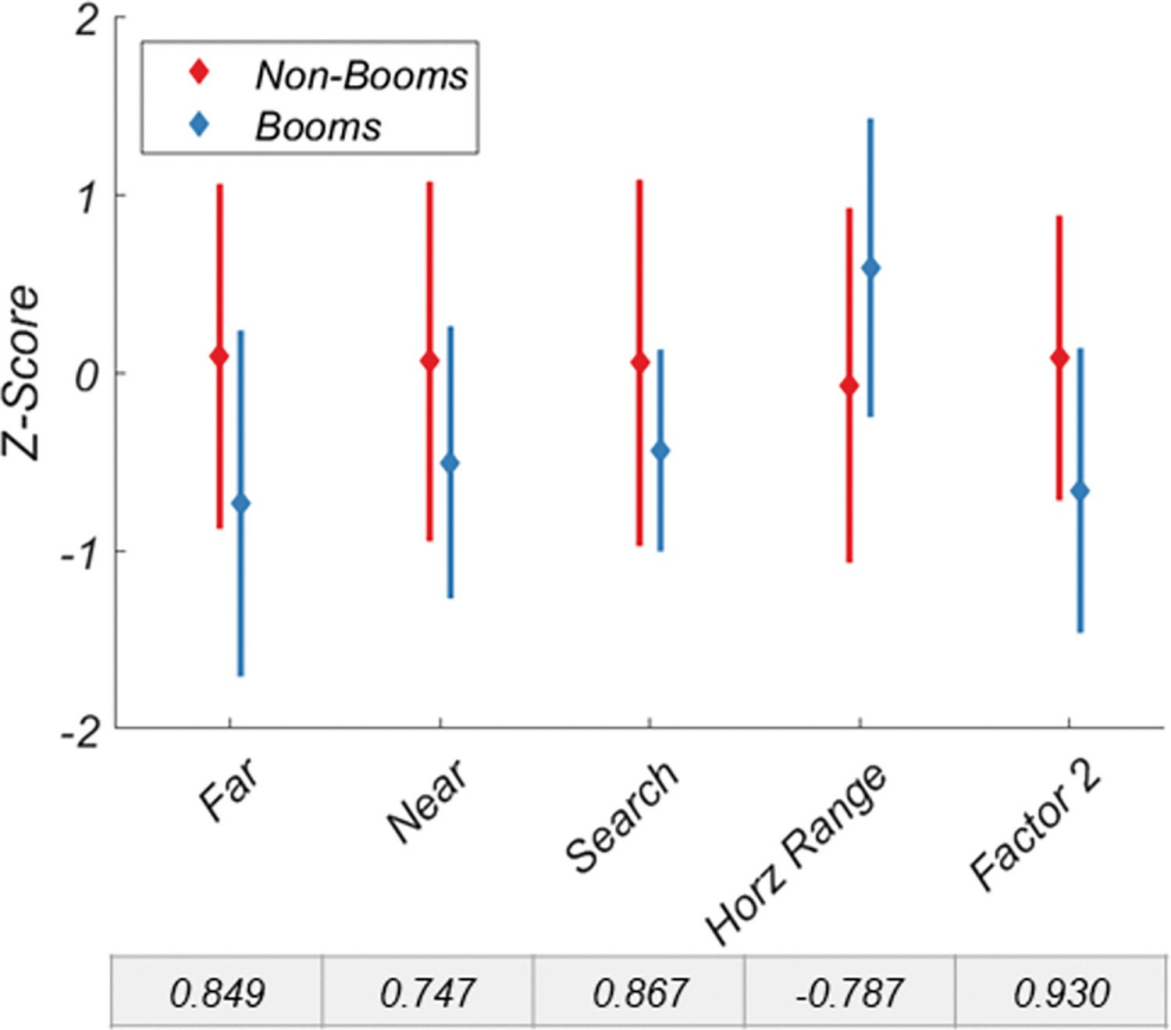
Discriminability of non-booms and booms for the binocular vision variables and factor 2. Data are normalized. Cohen’s D for each variable and factor is shown below each x-axis label. Error bars are +/- 1 SD.

**Fig 7:**
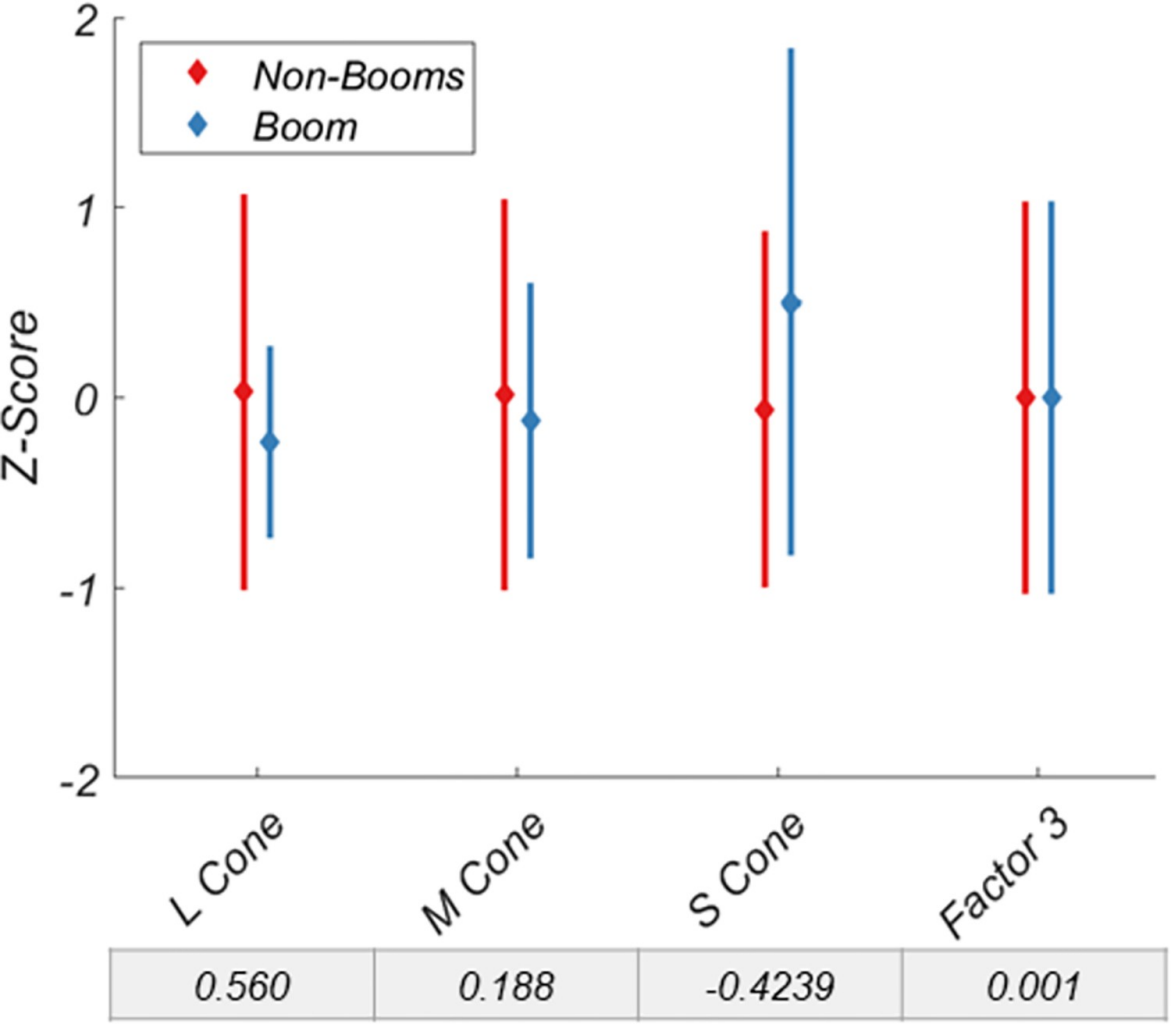
Discriminability of non-booms and booms for the color vision variables and factor 3. Data are normalized. Cohen’s D for each variable and factor is shown below each x-axis label. Error bars are +/- 1 SD.

**Fig 8:**
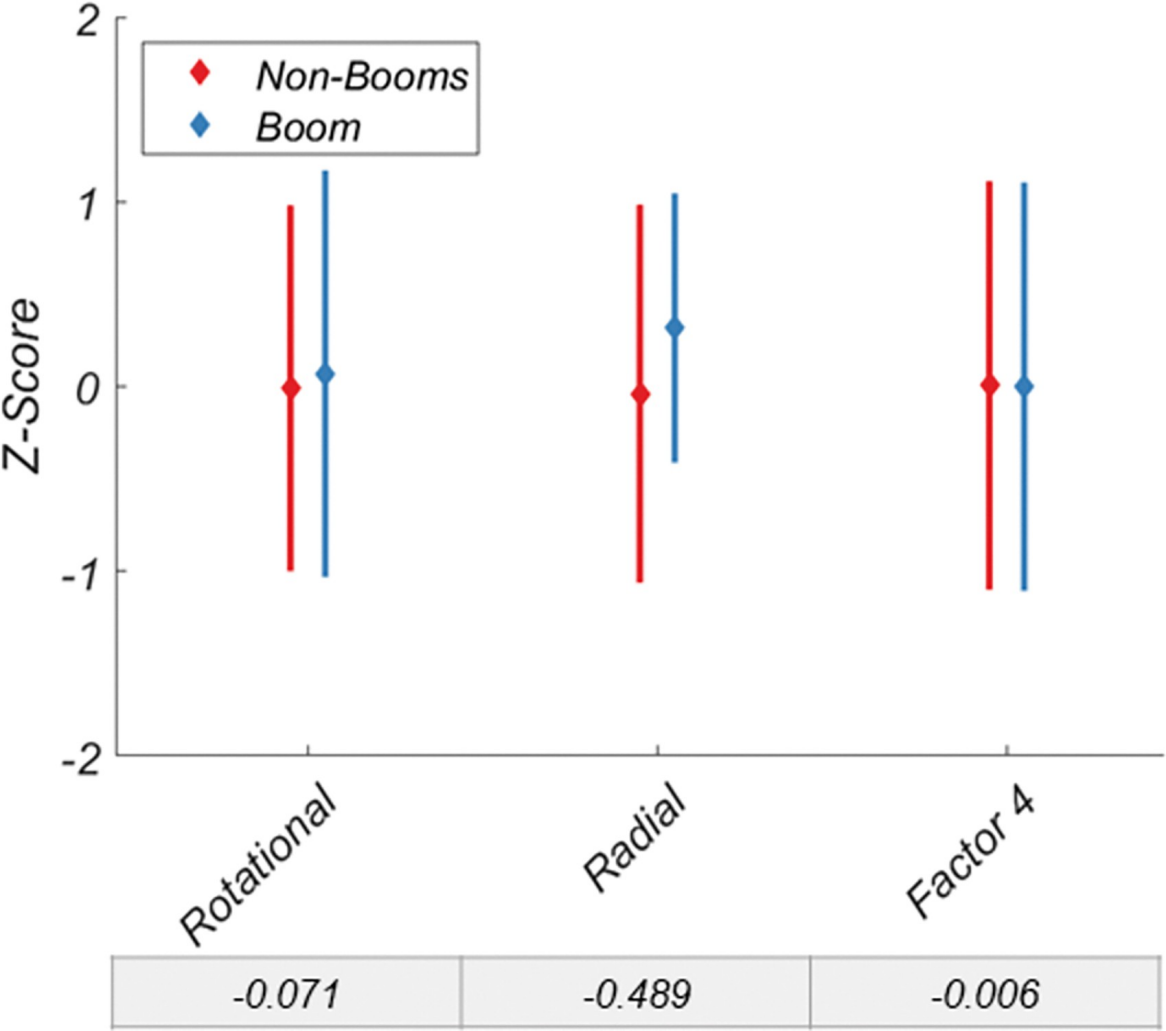
Discriminability of non-booms and booms for the motion coherences variables and factor 4. Data are normalized. Cohen’s D for each variable and factor is shown below each x-axis label. Error bars are +/- 1 SD.

A similar pattern is revealed when investigating the binocular vision variable (Fig 6), though no individual variable or factor exceeds a Cohen’s D of 1, Factor 2 outperformed any single variable at discriminating between the two groups. However, for color vision (Fig 7) and motion coherence (Fig 8), neither variables nor factors discriminated the two groups particularly well.

## Discussion

The results of this factor analysis suggest that there are 5 latent factors represented in the AVT dataset related to 1) higher SF vision, 2) binocular vision, 3) color vision, 4) motion perception, and (5) lower SF vision. The five factors account for 74% of the variance within this dataset. Though they are largely organized along predictable dimensions, the independence of higher and lower SF vision is curious but not without precedent. Similar approaches have been used to suggest the high and low SF vision develop independently and experience mature individual differences related to that independence [8,9]. These results, along with the data presented here, provide additional evidence for a broader SF channel processing hypothesis [28–31]. Further, the independence of low and high SF vision demonstrated here may be indicative of the further separation of the two channels in the dorsal and ventral stream, with higher SF content utilized in parvocellular mechanisms of the ventral “what” pathway and lower SF content utilized in the magnocellular mechanisms of the dorsal “where” pathway [32–34].

If it is indeed true that high and low SF information are processed relatively independently, then it may be likely that each have distinct naturalistic or operational application in functional vision. When comparing data from the general population and a visually demanding USAF occupation class, it does appear that discrimination between the two groups relies heavily on two independent mechanisms in spatial vision. Medium and high SF CS along with visual acuity are almost twice as discriminating between the groups as low SF CS, and the latent factor of higher SF vision outperforms all variables. This suggests that higher SF vision, but not lower SF vision, is more predictive of vocational success when specifically looking at boom operators in the USAF, suggesting a greater segregation of information in these two bandwidths. This is consistent with AVT data that shows a wider distribution of thresholds for the medium and high spatial frequency optotypes in comparison to the low spatial frequency optotype [3]. One caveat worth noting is that the Landolt C targets varied only by letter size and no attempt was made to bandpass their SF content; the largest optotype likely contained some high SF information. Despite this, the two channels still segregated largely independently.

Like Factor 1, Factor 2, the binocular vision factor, also had superior discriminability relative to its component variables. Factor 2 was correlated with near and far dual ring, and stereo search thresholds, along with horizontal fusional range. Interestingly, vertical fusional range was unrelated to Factor 2 (or any other factor). This may indicate that it is a truly independent mechanism, or it may lack variability within this dataset due to the limited range of vertical disparities to which the human visual system can respond [35–37]. Factors 3 (color vision) and 4 (motion coherence) were not good predictors of group membership. It is possible that the standards already in place to select active-duty boom operators may obviate their improved visual scores. However, the standards for Flying Class III among variables investigated here are 20/20 visual acuity, which is in the 93^rd^ percentile of the non-boom group (i.e., 93% have better visual acuity than the standard), and 40 arcseconds of stereoacuity, which is in the 63^rd^ percentile of the non-boom group. Under these standards, those with a stereoacuity of up to 120 arcseconds can receive a waiver and still pass, equivalent to the 80^th^ percentile of the control group. This would suggest that the standards themselves are not overly stringent and not self-selecting better visual statuses *a priori*.

An added applicable benefit of this approach is that it could help to streamline the vision screening battery. As it currently stands, the battery consists of 14 individual tests that, according to the results of this analysis, represent only 5 distinct latent factors. Thus, there is a substantial amount of redundancy among the tests in the current battery. If only one test were taken to represent each factor, the total time for the entire visual system snapshot could be reduced by 65% with only a limited loss of information. USAF Airmen participate in a demanding systematic medical screening that can take up to one week to complete. Although vision screening is only a portion of the required medical screening, the vision screening alone can also take a significant amount of time, particularly if repeated testing is required for some individuals. To streamline the testing involved, one could limit the battery to, for example, only low SF CS, acuity, stereopsis (near or far but not both), rotational motion coherence (or analogous global motion test), and a single color vision test, with limited loss of information. Of course this is posed only as a research question at this time and it should be noted that motion coherence is experimental only and not currently, and unlikely to be a vision standard anytime in the near future (though likely highly relevant to predicting real-world performance, as we have argued previously [38]). It is also worth noting that consideration should be given to the particular color vision test chosen. Though cone contrast sensitivities are associated with a single latent factor, the practical purpose of each is to identify specific independent color blindness patterns. Tritanopia, which is identified with the short-wavelength cone contrast test, is extraordinarily rare [39] and may be replaced with a quick and coarse screening test if time were of the essence. However, it seems likely that both medium- and long-wavelength cone contrast tests should remain, if only to satisfy the broader screening demands of the test.

The OBVA Lab is pursuing plans to develop a commercial version of the AVT. If successful, the commercial AVT will provide a unique platform to not only modernize occupational vision screening but will also provide a capability to support continued research concerning dimensions of vision and the relationship between vision and occupational and operational performance.
